# T-LysYal for Managing Dry Eye Disease, the Advent of Supramolecular Aggregates in Ophthalmology: A Narrative Review

**DOI:** 10.3390/jcm15020429

**Published:** 2026-01-06

**Authors:** Stefano Barabino, Marisa Meloni, Demetrio Manenti, Pauline Cipriano-Bonvin

**Affiliations:** 1 Ocular Surface & Dry Eye Center, ASST Fatebenefratelli SACCO, Milan University, 20157 Milan, Italy; 2 In Vitro Science and NAMs Expert, 20149 Milan, Italy; 3 Sildeha Swiss SA, 6900 Paradiso, Switzerland; 4 TRB Chemedica International SA, 1227 Carouge, Switzerland; pauline.cipriano-bonvin@trbchemedica.com

**Keywords:** dry eye disease, T-Lysyal, wound healing, ocular surface, supramolecular aggregate, nanotubes, hyaluronic acid

## Abstract

Dry Eye Disease (DED) is a highly characterised multifactorial disease resulting in the loss of tear film homeostasis and associated with a major impact on patient quality of life. DED affects up to half of the global population, with modern lifestyle factors playing a critical role in disease development, particularly excessive use of digital devices. The ultimate treatment goal is restoration of tear film homeostasis and breaking the ‘vicious circle’ of DED. Today, the use of tear substitutes represents the main option for the treatment of DED. These topical formulations aim to provide lubrication, reduce osmolarity, and improve tear clearance. However, they do not interact with the ocular surface epithelium nor modulate ocular inflammation, and do not fully restore natural tear function. T-LysYal is the first supramolecular ocular surface modulator for DED. Studies demonstrate that T-LysYal promotes tissue repair, improves tear breakup time, restores corneal epithelial cell damage, and modulates inflammation processes, significantly reducing the severity of DED symptoms in patients. In addition, T-LysYal provides stability that prolongs activity and favours cell adhesion. Through its 3D nanotube structure, movement of water in the eye is retained and improved, enhancing ocular hydrodynamics. This narrative review introduces T-LysYal for DED whilst highlighting both its in vitro activity and clinical profile against hyaluronic acid, a mainstay of disease management.

## 1. Introduction

Dry Eye Disease (DED) is poorly defined with a wide range of proposed disease subtypes, including tear film deficiencies, eyelid anomalies, ocular surface abnormalities, and systemic drivers [1]. The Tear Film and Ocular Surface Society Dry Eye Workshop II (TFOS DEWS II) epidemiology report on DED in 2017 suggests disease prevalence ranges from 5 to 50% of the global population; symptomatic disease may occur less frequently [2]. Nevertheless, DED is common and often symptomatic, affecting many millions worldwide [2]. Modern lifestyle factors play a role in the development and exacerbation of DED symptoms, with excessive use of digital devices as one of the most significant contributors [2,3].

DED is a multifactorial condition resulting in the loss of tear film homeostasis and progressing to the ‘vicious circle’ of DED: a cycle of tear film instability and hyperosmolarity in response to excessive evaporation, leading to inflammation [4,5] and consequently epithelial damage [5,6,7]. The ultimate goal of treatment is to restore tear film homeostasis by breaking the vicious circle that stimulates the disease [8].

Characteristic DED symptoms include ocular discomfort and visual disturbances, often accompanied by foreign body sensation, ocular dryness, redness, or excessive tearing [8]. Symptoms can have a major impact on both physical and psychosomatic wellbeing, with a significant impact on quality of life [9] driving an increased incidence of depression and anxiety in DED patients [3,8,10]. A diagnostic algorithm combining the abbreviated version of the Ocular Surface Disease Index questionnaire OSDI-6, tear film markers (non-invasive tear film breakup time, osmolarity) and ocular surface staining has been recently proposed by the TFOS DEWS III [1]. This refined diagnosis strategy should retain robustness while further simplifying its application in daily practice.

First-line treatment often focuses on methods to replenish, conserve, and stimulate the tear film [3]. Historically, tear substitutes have been used to provide lubrication to the ocular surface. Recent advances in the pathophysiology of DED clarified that treatment should focus not only on tear film quality or quantity, but also address the loss of ocular surface homeostasis and block the vicious circle of chronic inflammation and ocular damage [3]. However, tear supplements and stabilisers remain the cornerstone of DED management regardless of the underlying aetiology [3,11] and are easily accessible in a wide range of over-the-counter formulations [3]. Tear substitute products are classified as medical devices in most countries and must be supported by efficacy evidence and clinical trial outcomes [8].

Current tear supplements cover a wide range of formulation, including viscosity-enhancing agents, lipomimetic formulations, tear stabilisers, hyaluronic acid-based (HA) supplements, and hypo-osmotic or osmoprotective agents.

Viscosity-enhancing agents such as hydroxypropyl guar (HP-guar) bind to damaged hydrophobic regions of the ocular surface, forming a hydrated scaffold to protect and resist water loss [3]. When combined with polyethylene and/or propylene glycol, HP-guar-based supplements provide prolonged lubrication, significantly enhance tear film stability, reduce tear osmolarity, and improve overall ocular surface health [3].

Lipid-containing and lipomimetic products are often preferred in evaporative DED [12,13]. These formulations demonstrate efficacy through reductions in tear film surface tension, permitting an even distribution of tears over the ocular surface while decreasing tear evaporation, and reducing friction between the eyelid and ocular surface [14].

The tear stabiliser perfluorohexyloctane affects the lipid layer of the tear film and directly addresses excessive tear evaporation [15,16,17,18]. Topical perfluorohexyloctane appears to reduce corneal surface temperature, increasing the activity of cold thermoreceptors leading to an increased rate of blinking and lachrymation that may reduce ocular discomfort [19].

HA is a major component of the extracellular matrix, playing an important role in maintaining tissues, and is an increasingly common constituent of tear supplements [20,21]. HA has been associated with promoted wound healing [20], and high molecular weight HA provides anti-inflammatory and anti-apoptotic signals to cells exposed to UVB [21]. In preclinical models, HA has demonstrated a benefit for wound healing in corneal erosions [20].

It is important to highlight that generally available tear substitute products are often designed to mimic the tear film but often lack the biologically active components found naturally in situ [3,14]. In addition, the overall formulation and composition of such products may affect their performance, as efficacy is not solely related to individual ingredients [3]. Overall, there is a paucity of tailored, long-lasting, preservative-free products available, with a general failure to directly address tear film instability and the underlying causes of DED. An absence of robust efficacy comparisons across existing treatments may also further obfuscate appropriate treatment selection. 

Availability of a new class of agents termed ‘ocular surface modulators’ may offer a novel approach to tear film homeostasis and restoration of damaged corneal cells in DED [8]. Ocular surface modulators comprise polymers with the capability to influence ocular surface components by promoting restoration of corneal cells, improving tear film homeostasis, and modulating the inflammatory process [8].

T-LysYal is a supramolecular compound containing lysine and thymine hyaluronate, and sodium chloride that forms a 3-D structure with nanotubes [22]. While HA binds water, the T-LysYal compound attracts water and also has the capacity for improving ocular hydrodynamics [23] and modulating the recruitment of inflammatory cells [23,24].

In addition, T-LysYal is more resistant to the lytic enzyme hyaluronidase than HA, providing superior product stability that may enable a longer activity period, favouring cell adhesion, differentiation, and secretion [25,26]. In vitro, T-LysYal has demonstrated the ability to repair corneal epithelial cells damaged by dry conditions [26].

The aim of the present narrative review is to examine T-LysYal by briefly investigating its mechanism of action and highlighting its potential benefits in DED. T-LysYal has been proposed to be the first ocular surface modulator, and elucidating its properties might open new possibilities in the future management of DED.

## 2. T-Lysyal in Wound Healing

Impaired wound healing has been proposed to be tightly associated with DED pathology. Recent work confirmed that the underlying inflammation observed in DED is associated with delayed corneal repair [27,28]. Information gained from other indications on the impact of T-LysYal on tissue repair is therefore of interest to better understand the expected benefits of its use in DED.

Administration of T-LysYal has shown promising results in tissue regeneration, improving the healing process in decubitus ulcers and burn injuries [29,30], rapidly restoring nasal mucosa after functional endoscopic sinus surgery [24,31], and repairing in vitro cells damaged by Dry Eye Disease modelling [26].

Felzani et al. (2011) investigated the use of T-LysYal in a randomised, double-blind study of 50 chronically hospitalised patients with a range of ulcer severities (erythema and oedema; all-thickness skin destruction; destruction of subcutaneous tissue) [29]. Application of T-LysYal was associated with a superior reduction in lesion size across all ulcer groups over a 15-day period compared with sodium hyaluronate [29]. Time to 50% lesion regression was also significantly reduced [29]. More recent case study reports by Haydar et al. (2023) highlight the potential value of T-LysYal in a cream formulation for wound healing and pain relief from superficial partial-thickness burn injuries [30].

Further evidence of a role in wound healing comes from nasal studies, where T-LysYal has been investigated in both rhinitis (as a replacement for concomitant HA), and following functional endoscopic sinus surgery (FESS) [24,31].

As ancillary therapy in patients with allergic, non-allergic, and mixed rhinitis, T-LysYal was associated with significant reductions in symptoms and endoscopic features [24]. In addition, the grades of both eosinophilic and neutrophilic infiltrates were reduced over a 4-week treatment period, providing a profound impact on mucosal repair [24]. In patients receiving prior FESS, adjunctive intranasal treatment with T-LysYal significantly reduced symptoms, endoscopic features, and inflammatory cells over a 4-week follow-up period compared with isotonic saline alone [31].

## 3. T-Lysyal in Dry Eye Disease

### 3.1. Preclinical Evaluation

Utilising an in vitro model of DED based on human corneal epithelium submitted to hyperosmotic conditions to evaluate the potential effects of T-LysYal on damaged corneal epithelial cells, Barabino and colleagues demonstrated that treatment over 24 hours following a dry eye stress challenge produced significant improvements in ultrastructural morphology observed under scanning electron microscopy [26].

**Figure 1:**
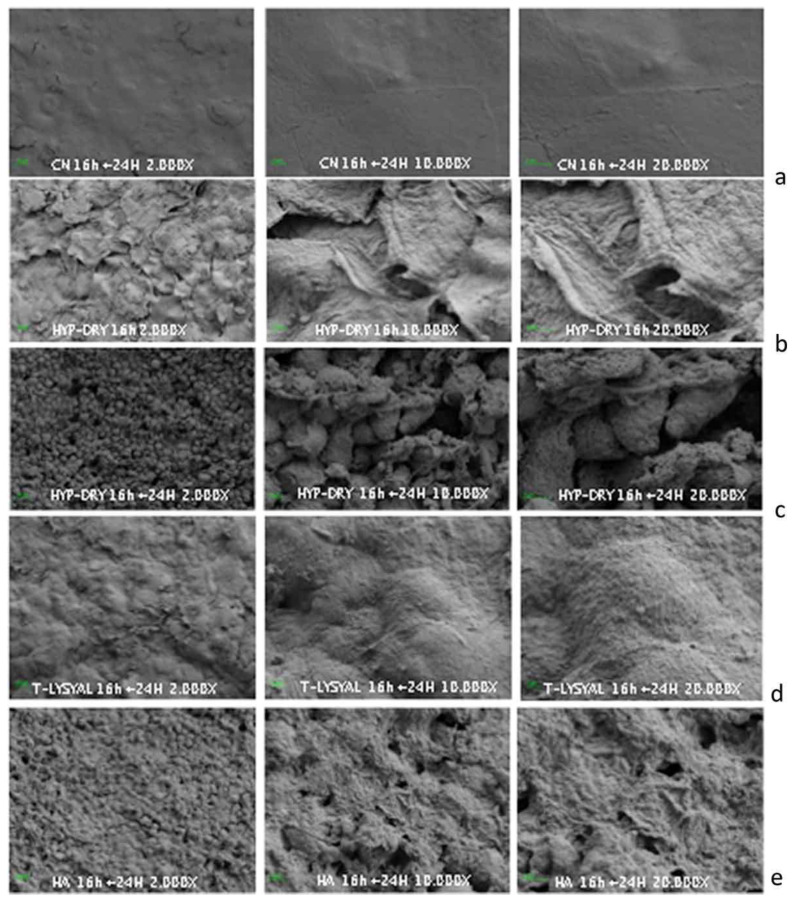
Impact of T-LysYal on the structure of human corneal epithelium ex vivo. Scanning electron microscopy images of human corneal epithelium in standard, HYP-DRY condition (dry hyperosmolar conditions), and after treatment with T-LysYal and hyaluronic acid (HA). Magnification: 2000–10,000–20,000×. (**a**) Human corneal epithelium in standard conditions: the cell surface appears smooth and covered by microvilli. (**b**) After 16 h in HYP-DRY condition the surface of human corneal cells appears irregular with visible dehydration and reduction in cell-to-cell connections. (**c**) A similar picture was visible after 24 h recovery post stress. (**d**) After 24 h of treatment with T-LysYal, the cell surface appears restructured with an ultrastructure similar to cells in standard conditions. (**e**) Cells treated with HA for 24 h show a limited recovery compared to cells treated with T-LysYal. Reproduced with permission from [26].

The Dry Eye Disease model is based on reconstructed human corneal epithelium (HCE) damaged by severe osmotic stress and mimics the changes that occur in the immunocompetent dry eye [32]. These include inflammation and enhanced expression of pro-inflammatory cytokines and chemokines, along with the infiltration of autoreactive T cells responsible for disease chronicity [32]. Following T-LysYal, the HCE surface presented an ultrastructure similar to the negative control (90% humidity, 37 °C, and 5% CO_2_), with observed cell surfaces being smooth and without visible extracellular matrix protein structures (Figure 1) [26].

The active role of T-LysYal in this model is demonstrated by the modulation of integrin-β1 (involved in cell-to-cell contacts) allowing epithelial cells to adhere to each other and promote tissue repair [26]. After 24 hours of treatment, there was an increased expression of integrin-β1 in the corneal tissue vs. control. In the control group, treatment with HA induced a limited, positive recovery of the tissue surface ultrastructure, but was unable to reproduce the changes induced by T-LysYal [26].

During the development of the HCE model by Meloni and colleagues, THP-1 (human leukaemia monocytic cell line) infiltration was monitored via immunohistochemistry and molecular biology over 24 hours following application of T-LysYal. Expression of CD14, CD86, and aquaporin-3 (both gene and protein) was reduced [23]. The transmembrane water channel protein aquaporin-3 (AQP-3) is distributed in the lacrimal gland, cornea, and conjunctiva [33]. AQP-3 is associated with water transport across cell membranes in response to osmotic gradients and is usually over-expressed in DED, reflecting the high levels of membrane water permeability observed in this disease [23]. By inducing down-regulation of AQP-3 gene and protein expression, T-LysYal was associated with a rebalancing of water flux regulation at a molecular level, and thus an improvement of ocular hydrodynamics [23].

**Figure 2:**
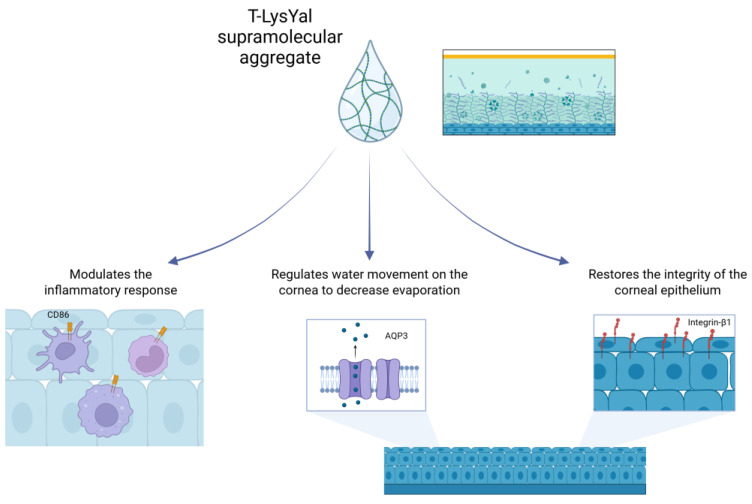
Summary of T-LysYal mechanism of action. Created in BioRender.com.

Outcomes from the HCE model suggest that T-LysYal significantly improves the conditions of the corneal epithelium following damage in dry eye conditions by modulating tissue growth factor expression and restoring the integrity of epithelial cells [26] (Figure 2).

### 3.2. Clinical Evaluation

In 2025, Barabino et al. performed a randomised, double-blind pilot study in patients with DED symptoms treated with T-LysYal (*n* = 12) or HA 0.2% + tamarind seed polysaccharide (control group, *n* = 15) for 2 months [34]. The aim of the evaluation was to determine whether T-LysYal improved the symptoms and signs in patients with DED [34].

**Figure 3:**
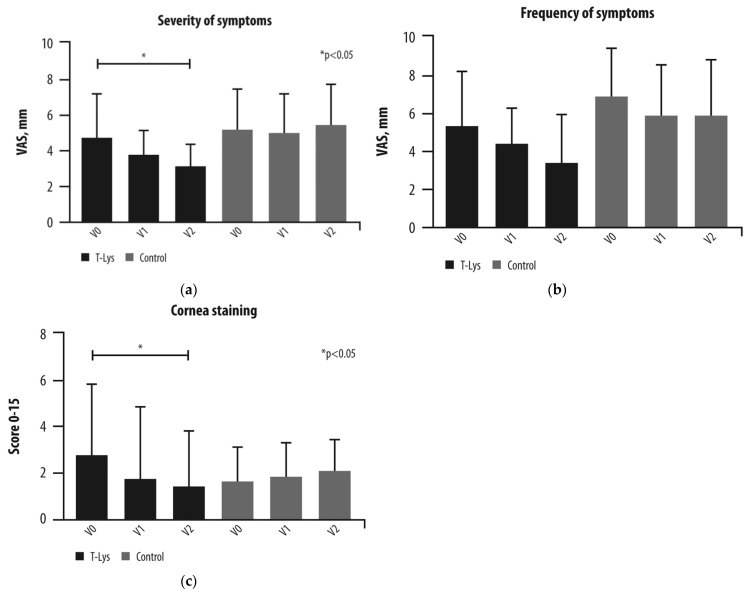
Clinical evaluation of 0.32% T-LysYal eye drops in patients with Dry Eye Disease. (**a**) The severity of symptoms was statistically lower after 2 months in comparison to the baseline in the T-LysYal group. * *p* < 0.05. The *Y*-axis unit is a visual analogue scale, i.e., a scale to measure the characteristics of the pain of the patient. (**b**) Frequency of symptoms did not change through the treatment in both the T-LysYal and control group. The *Y*-axis unit is a visual analogue scale, i.e., a scale to measure the characteristics of the pain of the patient. (**c**) Damage of the cornea epithelium decreased after 2 months in the T-LysYal group (*p* < 0.05). V0 represents baseline (before treatment), V1 represents 1 month after treatment, and V2 represents 2 months after treatment. Reproduced with permission from [34].

There were significant improvements from baseline to 2 months in the severity of DED symptoms following T-LysYal, as assessed by SANDE (Symptom Assessment in Dry Eye) Visual Analogue Scale (Figure 3) [34]. Symptom severity did not differ between baseline and month 2 in the control group, and symptom frequency was not significantly changed between groups [34].

Corneal staining was used to assess damage to the corneal epithelium, and significant improvements were observed from baseline following T-LysYal; there was no change in cornea staining following HA [34]. However, there were no statistical differences between the two groups at the 1- or 2-month assessment visits. Tear film breakup time was significantly higher following 2 months of T-LysYal but did not change following HA [34].

Limitations to the study include a relatively small sample size (*n* = 27) and lack of long-term follow-up [34]. Tear volume, quality of lipid layer, and non-invasive tear breakup time were not assessed in this pilot study [34]. However, these study outcomes support the preclinical and modelling data in DED and provide the first clinical evidence of efficacy in patients, highlighting the potential of T-LysYal as an ocular surface modulator [34].

## 4. Discussion

Since May 2021, the European Union Medical Devices Regulation (Regulation 2017/745) (EU MDR) has applied in EU Member States and Northern Ireland [35]. As tear substitutes are classified as medical devices within the EU, the regulation also applies to DED therapies, requiring new tear substitute products to be accompanied by scientific proof of efficacy and a clinical trial at least 1 year from product launch [35]. The same rules apply to tear substitutes currently available, for which new and more robust documentation would be required [8,35].

Topical tear substitutes are a favoured treatment for DED symptoms [3,8], although interpretations of relative efficacy and safety are constrained by variabilities in study methodologies, patient populations, and corneal staining grading scales, as well as the absence of direct head-to-head treatment comparisons [36,37]. In addition, very few therapies provide an opportunity to directly improve damage to ocular surface epithelial cells associated with DED [8].

Lifestyle modifications, including optimised blinking, dietary supplementation, and environmental adjustments, play a crucial role in long-term management of DED [3]. These must be combined with patient education regarding management, treatment, and prognosis, which remain essential for treatment adherence and sustained symptom relief [3].

T-LysYal is an ocular surface modulator based on a supramolecular aggregate that affects the inflammatory process in DED [34] and improves the ultrastructural organisation of corneal epithelium [23]. Clinical outcomes suggest T-LysYal is associated with significant improvements over 2 months in patient symptoms, corneal staining, and tear film breakup time [34] (Table 1).

## 5. Conclusions

This narrative review summarises the actual knowledge regarding the mechanism of action of T-LysYal and the expected benefits for patients suffering from DED. T-LysYal promotes tissue repair [8,26,30] and significantly reduces the severity of DED symptoms in patients [34]. In addition, T-LysYal may provide resistance to hyaluronidase degradation with an associated stability that prolongs activity and favours cell adhesion, differentiation and secretion [22,25]. 

T-LysYal is the first supramolecular aggregate ocular surface modulator for DED, which retains and improves the movement of water [24] associated with ocular hydrodynamics [23].

## Figures and Tables

**Table 1:** T-LysYal key points summary.

T-Lysyal
First supramolecular aggregate ocular surface modulator for DED, retaining and improving the movement of water [24] associated with ocular hydrodynamics [23]
Promotes tissue repair via integrin-β1 modulation [8,26,30]
Significantly reduces the severity of DED symptoms in patients [34]
Improves ultrastructural morphology in a DED model [26]

## Data Availability

No new data were generated for this publication.

## Abbreviations

The following abbreviations are used in this manuscript:

DED	Dry eye disease
HA	Hyaluronic acid
